# Effects of Multispecies Synbiotic Supplementation on Anthropometric Measurements, Glucose and Lipid Parameters in Children With Exogenous Obesity: A Randomized, Double Blind, Placebo-Controlled Clinical Trial (Probesity-2 Trial)

**DOI:** 10.3389/fnut.2022.898037

**Published:** 2022-07-01

**Authors:** Gonca Kilic Yildirim, Meltem Dinleyici, Yvan Vandenplas, Ener Cagri Dinleyici

**Affiliations:** ^1^Department of Pediatrics, Faculty of Medicine, Eskisehir Osmangazi University, Eskisehir, Turkey; ^2^Pediatrics Nutrition and Metabolism Unit, Faculty of Medicine, Eskisehir Osmangazi University, Eskisehir, Turkey; ^3^Department of Social Pediatrics, Faculty of Medicine, Eskisehir Osmangazi University, Eskisehir, Turkey; ^4^UZ Brussel, KidZ Health Castle, Vrije Unversiteit Brussel, Brussels, Belgium

**Keywords:** obesity, children, adolescent, probiotic, synbiotic

## Abstract

Studies on the effects of synbiotics on obesity in children are limited. The objective of this randomized double-blind placebo-controlled trial was to test the effects of a multispecies synbiotic during 12 weeks on anthropometric measurements, glucose metabolism and lipid parameters in 61 children with exogenous obesity. All children were treated with a standard diet and increased physical activity and received once daily a synbiotic supplement (probiotic mixture including *Lactobacillus acidophilus, Lacticaseibacillus rhamnosus, Bifidobacterium bifidum, Bifidobacterium longum, Enterococcus faecium* and fructo-oligosaccharides) or daily placebo for 12 weeks. At baseline, no statistically significant differences existed in anthropometric measurements, glucose and lipid parameters between both groups. We observed changes for anthropometric measures (% reduction comparing to baseline) in both synbiotic and placebo groups. After 12 weeks; changes (% reduction comparing to baseline) in weight (*p* < 0.01), BMI (*p* < 0.05), waist circumference (*p* < 0.05) and waist circumference to height ratio (*p* < 0.05) were significantly higher in the children receiving the synbiotic supplement. There is no difference in glucose metabolism, lipid parameters, presence of non-alcoholic fatty liver disease between both groups after 12 weeks. The daily intake of a multispecies synbiotic in addition to diet and increased physical activity did improve anthropometric measurements: body weight, BMI, waist circumference and waist/height ratio. The supplementation of this synbiotic is an efficient weight-loss strategy above diet and exercise in pediatric obesity (Trial identifier: NCT05162209).

## Introduction

Obesity is a critical public health concern that affects ~20% of the world's population and is linked to a number of serious comorbidities including metabolic, cardiovascular, respiratory, and cancer illnesses in both developed and developing countries (1, 2). An estimated 38 million children under the age of five years and over 340 million children and adolescents aged 5–19 years were overweight or obese. The prevalence of overweight and obesity among children was 18% in 2016 (1). Childhood obesity is classified as exogenous or endogenous, depending on the etiology. Exogenous obesity is produced by a long-term imbalance in energy intake and expenditure, whereas endogenous obesity is caused by a variety of genetic, syndromic, and endocrine factors (3). The majority of obese children and adolescents grow up to be obese adults. Obesity in childhood not only leads to long-term health issues that manifest in adulthood, but it also leads to secondary complications such as dyslipidemia, insulin resistance, and non-alcoholic fatty liver disease (1, 2, 4, 5). Obesity in children is typically treated by reducing energy intake through food regulation and increasing energy expenditure through increased activity (2).

Many disorders (obesity, metabolic syndrome, diabetes, asthma, and atherosclerosis) have been demonstrated to have altered microbiota compositions (dysbiosis), but research is currently ongoing to identify whether these changes are cause or effect. As a result, it's suggested that manipulating the gut microbiota could be a therapeutic target for reducing host energy storage (4). There have been studies on the use of probiotics and prebiotics as a support for obesity treatment, however the majority of these studies involved adults (6, 7). The synbiotic is a mixture, comprising live microorganisms and substrate(s) selectively utilized by host microorganisms, that confers a health benefit on the host, according to the International Scientific Association of Probiotics and Prebiotics. Complementary and synergistic synbiotics are the two types of synbiotics (8). Probiotics and synbiotics, specifically certain strains of *Lactobacillus gasseri, L. rhamnosus, and L. plantarum*, associated with other *Lactobacillus species* and/or species from the Bifidobacterium genus, have the potential to aid in weight and fat mass loss in overweight and obese populations, according to the systematic review (5). *L. acidophilus* in combination with *L. casei and Bifidobacterium*, or *L. acidophilus* in combination with *Bifidobacterium infantis*, had positive benefits on body weight loss in participants who maintained their usual lifestyle (9, 10). It has also been reported that daily ingestion of diet-enriched prebiotics, such as fructooligosaccharide, enhances satiety. However, while experimental studies indicate the positive effect of prebiotics in obesity, clinical trial outcomes are mixed (11). The standard treatment of obesity in children is based on a reduction of the energy intake by regulating the diet and increasing the energy expenditure by increasing the activity. There are studies on the use of probiotics and prebiotics as a support for treatment in obesity, but most of these studies were conducted in adult age groups. Studies on the effects of synbiotics on obesity in children are limited (12, 13). The goal of this randomized, double-blind, placebo-controlled trial was to see how this particular multi-strain synbiotic affected anthropometric measurements, glucose metabolism, and lipid markers in children with exogenous obesity.

## Patients and Methods

### Study Design

This is a single-center, prospective, randomized, double-blind, placebo-controlled clinical study in children aged between 8 and 17 years with exogenous obesity who admitted for the first time to Eskisehir Osmangazi University Faculty of Medicine, Department of Pediatrics, Nutrition and Metabolism Department between January 2019-June 2021. This clinical study was planned and performed in accordance with the Declaration of Helsinki and Good Clinical Practice guidelines, patient rights regulation and ethical committees. Permission for the study was obtained from the Clinical Research Ethics Committee of Eskisehir Osmangazi University Faculty of Medicine with the Decision Number 54 on September 27, 2018. This study is registered in ClinicaTrials.gov under the Identifier number NCT05162209. The study protocol was explained to all participants and their families, and a written informed consent was obtained from all parents and children prior to the inclusion.

### Study Population, Inclusion and Exclusion Criteria

**Figure 1:**
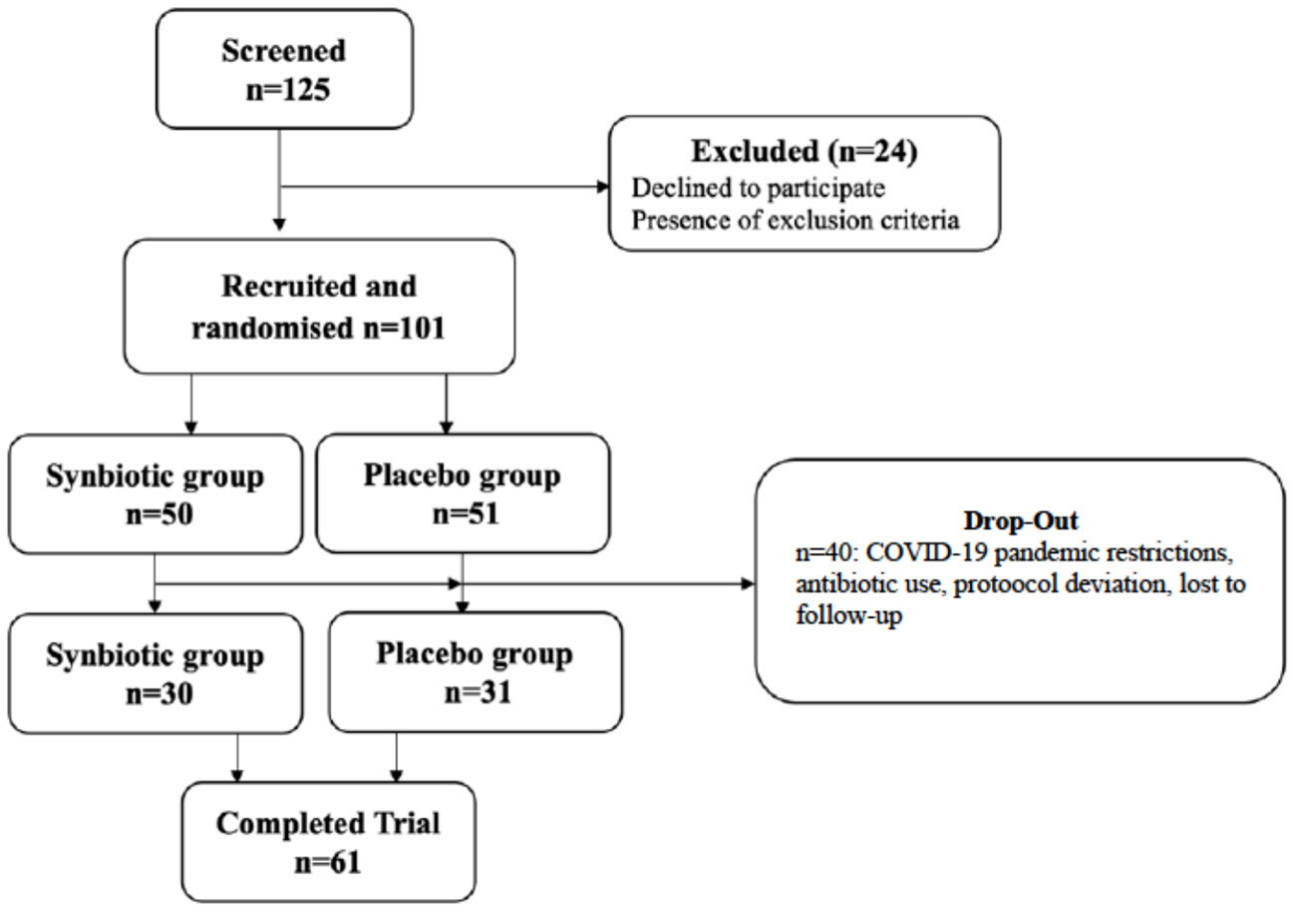
Flow chart of the study.

Children and adolescents, aged 8–17 years, with a body mass index (BMI) equal to or higher than the age- and sex-specific 95^th^ revised percentiles of the Centers for Disease Control and Prevention (CDC) were evaluated according to the study criteria (14). Patients who had no pathological findings other than obesity in their physical examination were considered as “exogenous obese” and included in the study (3). Patients with secondary obesity or endogenous obesity, history of any chronic diseases and/or chronic medication use and/or monogenic syndromes and other genetic syndromes, or those under special diets were excluded from the study. Patients who used probiotics/synbiotics/fibers or antibiotics in the 8 weeks before possible inclusion, were also excluded. The flow chart of the study shown in Figure 1.

### Physical Examination, Anthropometric Measurements and Calculation

The participants' age and gender were recorded. A detailed nutritional history of the patients was collected. A full physical examination (including blood pressure measurement, presence of striae and acanthosis nigricans) have been performed. All anthropometric measurements (body weight, height, waist and hip circumference) were made by a trained nurse using standard protocols and calibrated instruments. Weight and height of participants were measured with light clothes and without shoes. Body weights were measured with a classical scale and height was measured in centimeters, standing upright, using a wall mounted stadiometer. CDC data were used to evaluate body weight and height measurements (14). BMI was calculated as the ratio of body weight (kilograms) to height squared (meter square). The circumference of the middle arm was measured at the left arm at the middle of the distance between shoulder and elbow; skinfold thickness was measured at the midpoint of the left shoulder and elbow over the triceps using a skinfold caliper. Waist and hip circumferences were measured with a non-elastic tape. Waist circumference was measured while the child was standing, with the abdomen relaxed, arms at the sides, and feet together, by wrapping a non-elastic tape measure around the thinnest part of the body, at a point midway between the lower border of the rib cage and the iliac at the end of expiration. The hip measurement was taken by holding it parallel to the ground, at the maximum girth of the buttocks. Waist-to-height and waist-to-hip ratio was calculated.

### Laboratory Tests

Laboratory tests including serum glucose, insulin, serum liver markers (aspartate aminotransferase-AST; alanine aminotransferase-ALT, serum lipid parameters including triglyceride (TG), total cholesterol (TC), high-density lipoprotein-cholesterol (HDL-C), low-density lipoprotein-cholesterol (LDL-C) after following 12 h of fasting from all patients have been noted at baseline and 12 weeks later. The diagnosis of dyslipidemia was made according to the criteria ≥95th percentile of each serum TG, TC, LDL-C level or below 5th percentile of HDL-C level by comparing with reference values according to age and gender (15). The degree of insulin resistance was estimated with the homoeostatic model assessment for insulin resistance (HOMA-IR) score was calculated with the formula (glucose) x (insulin)/405. The diagnosis of non-alcoholic fatty liver disease (NAFLD) was based on ultrasonographic findings and sex-specific ALT reference ranges (normal ALT <26 U/L in males and <22 U/L in females) (16).

### Diet and Increasing the Physical Activity

The definition of obesity, its effects on the body, complications and the course of the intervention were explained in detail to the patients and their families during about 30 min. A dietary intervention and increased physical activity were recommended in all cases. All obese children recorded their daily food consumption during last three days. The diets of the patients were reduced with 10% from their habitual caloric intake. No foods were banned, but cutting back on high-energy foods and drinks were recommended. The total energy content was composed so that 55% came from complex carbohydrates, 30% from fats and 15% from proteins. Daily cholesterol intake was regulated not to exceed 300 mg. The intake of saturated fats was planned to be <10% of the total energy intake and the intake of trans fats was planned to be <1% of the total energy intake. The intake of total polyunsaturated fatty acids was limited to 15%. Free sugar intake was reduced below 10% of total energy intake. All enrolled children monitored *via* a phone call once per 15 days and the patients were called to the outpatient clinic controls once a month. The diet was checked according to the verbal statements of the individual about the diet menus prepared for the individual. In addition, the declared dietary content was calculated by the same dietitian at each visit. In addition to their normal activities, the patients were advised to exercise moderate and heavy for at least 30 min daily. At each visit, they were questioned about their compliance with the exercise.

### Randomization, Intervention and Masking

The patients were divided in two groups by a computer-generated randomization sequence which assigned participants in a 1:1 allocation ratio to treatment with synbiotic or placebo with blocks of 8, blinding the study team, patients and their relatives. Interventional products were numbered, and all investigators and patients were blinded for all the duration of the study. Treatment duration was 12 weeks. In the first group, 1 sachet each day for 12 weeks (*Lactobacillus (L.) acidophilus* (4.3x10^8^ CFU/sachet), *Lacticaseibacillus (L.) rhamnosus* (4.3 x 10^8^ CFU/sachet), *Bifidobacterium (B.) bifidum* (4.3x10^8^ CFU/sachet), *B. longum* (4.3x10^8^ CFU/ sachet), *Enterococcus faecium* (8.2 x 10^8^ CFU/sachet), total 2.5 x 10^9^ CFU per sachet, fructooligosaccharide (FOS) 625 mg, lactulose 400 mg, Vitamin A (6 mg), Vitamin B1 (1.8 mg), Vitamin B2 (1.6 mg), Vitamin B6 (2.4 mg), Vitamin E (30 mg), Vitamin C (75 mg) were given. The second study group was given a placebo consisting of a similar sachet with shape, taste, and smell identical to the synbiotic sachet for 12 weeks.

### Follow-Up

Anthropometric measurements and biochemical indices were evaluated in all participants at baseline and after 12 weeks of intervention. Study compliance was monitored *via* a phone call once per 15 days and the patients were called to the outpatient clinic controls once a month. The patients were asked to record and contact the study team in case of an undesirable effect associated with the use of interventional products. Patients were asked to report if antibiotic therapy was started during the study; these children were also excluded from the study.

### Outcomes

The primary outcome of the study was to evaluate the effects of synbiotics on the anthropometric measurements after 12 weeks. Secondary end points were the effects of synbiotics on lipid parameters, presence of hyperlipidemia, glucose metabolism, and NAFLD.

### Statistical Analysis

Statistical Package for Social Sciences (SPSS) version 28.0 for Windows (SPSS - Chicago, IL, United States) was used for statistical analysis. Statistical analyses were performed according to per protocol. Continuous variables were expressed as mean, given as standard deviation. After assessment of the normal distribution by the Kolmogorov-Smirnov test, anthropometric measurements, glucose and lipid parameters were compared among the groups by using the independent sample *t*– test for continuous data and a chi-square test for categorical data. Paired Student's *t*-tests were used for comparing baseline and 12 weeks of intervention in synbiotic and placebo group. *p*-Values lower than 0.05 were considered as statistically significant.

## Results

Data from 61 children; 33 girls (18 synbiotic/15 placebo) and 28 boys (12 synbiotics; 16 placebo), aged between 8 and 17 years with exogenous obesity, who completed the 12 study-weeks (30 in the placebo group and 31 in the placebo group) were available. There was no statistically significant difference in gender (*p* > 0.05). The mean age of the patients in the synbiotic group was 11.8 ± 3.1 years, and 12.4 ± 2.7 years in the placebo group (*p* > 0.05). The anthropometric parameters of the synbiotic and placebo group at baseline and at the end of the 12th weeks of intervention are provided in Table 1.

**Table 1:** Evaluation of the change in anthropometric measurements of the synbiotic and placebo groups at the beginning of the study and at the end of the 12th week.

**Parameters**	**Synbiotic group** ***n*** **=** **30**				**Placebo group** ***n*** **=** **31**				**p3**
	**Baseline**	**12^**th**^ Weeks**	**% Reduction**	**p1**	**Baseline**	**12^**th**^ weeks**	**% Reduction**	**p2**	
Weight (kg)	67.6 ± 18.6	64.4 ± 18.3	4.0 ± 3.1	***p** **<** **0.001***	75.4 ± 23.1	74.2 ± 21.9	1.2 ± 4.19	ns	***p** **<** **0.01***
Weight z-score	2.12 ± 0.37	1.97. ± 0.36	8.7 ± 7.3	***p** **<** **0.001***	2.22 ± 0.75	2.08 ± 0.79	5.1 ± 6.5	***p** **<** **0.01***	***p** **<** **0.05***
Height (cm)	152.3 ± 14.1	153.1 ± 14.0	-	**ns**	157.2 ± 11.8	158.3 ± 11.5	-	ns	ns
Weight-for-Height (%)	151.2 ± 16.1	146.4 ± 14.7	3.0 ± 3.9	***p** **<** **0.01***	149.1 ± 18.9	148.9 ± 20.3	0.3 ± 0.7	ns	ns
BMI (kg/m^2^)	28.2 ± 3.7	26.7 ± 3.7	5.1 ± 3.1	***p** **<** **0.001***	29.8 ± 6.0	29.0 ± 5.8	1.1 ± 3.4	***p** **<** **0.05***	***p** **<** **0.01***
BMI Z-score	2.06 ± 0.26	1.88 ± 0.28	8.78 ± 7.31	***p** **<** **0.001***	2.08 ± 0.39	2.04 ± 0.43	5.1 ± 6.56	***p** **<** **0.05***	***p** **<** **0.001***
TSFT (mm)	28.2 ± 5.3	24.5 ± 6.3	13.6 ± 13.9	***p** **<** **0.01***	28.6 ± 7.4	26.5 ± 6.8	6.1 ± 16.7	***p** **<** **0.05***	ns
UAL (cm)	30.4 ± 3.4	29.0 ± 3.3	4.4 ± 4.3	***p** **<** **0.001***	32.7 ± 5.7	31.5 ± 5.68	3.4 ± 6.0	***p** **<** **0.01***	ns
Waist circumference (cm)	92.5 ± 8.5	86.8 ± 8.7	6.0 ± 4.8	***p** **<** **0.001***	96.7 ± 15.0	93.1 ± 14.7	3.7 ± 3.4	***p** **<** **0.001***	***p** **<** **0.05***
Hip circumference (cm)	107.0 ± 14.4	104.0 ± 13.8	2.7 ± 3.5	***p** **<** **0.001***	102.6 ± 12.7	98.8 ± 12.1	3.6 ± 3.7	***p** **<** **0.001***	ns
Waist/height ratio	0.60 ± 0.03	0.56 ± 0.04	4.43 ± 3.25	***p** **<** **0.001***	0.61 ± 0.03	0.56 ± 0.03	6.58 ± 4.77	***p** **<** **0.001***	***p** **<** **0.05***
Waist/hip ratio	0.90 ± 0.02	0.88 ± 0.06	2.44 ± 4.90	ns	0.90 ± 0.06	0.89 ± 0.07	0.89 ± 5.11	ns	ns

*The data were expressed as mean ± standart deviation. p1: Baseline vs. 12^th^ week in synbiotic group, p2, Baseline vs. 12^th^ week in placebo group; p3: Synbiotic group vs. Placebo group at 12 weeks. BMI, body mass index; TSFT, triceps skinfold thickness; UAL, upper arm length.*

*Bold values are statistically significant*.

### Comparison of Anthropometric Measurements

At baseline, there was no statistically significant difference between the two groups for anthropometric measurements and calculations (*p* > 0.05).

In the synbiotic group, at the end of the 12^th^ week, all the following parameters were lower compared to baseline: body weight (*p* < 0.001), body weight Z-score (*p* < 0.001), BMI (*p* < 0.001), BMI Z-score (*p* < 0.001), triceps skinfold (*p* < 0.001) thickness (*p* < 0.001), upper arm circumference (*p* < 0.001), waist circumference (*p* < 0.001), hip circumference (*p* < 0.001), and waist-to-height ratio (*p* < 0.001) were lower.

In the placebo group, compared to baseline, at the end of the 12th week, there was no statistically significant decrease in body weight (*p* > 0.05), while body weight Z-score (*p* < 0.05), BMI (*p* < 0.05), BMI Z-score (*p* < 0.01), triceps skinfold thickness (*p* < 0.05), upper arm circumference (*p* < 0.05), waist circumference (*p* < 0.01), hip circumference (*p* < 0.01), and waist-to-height ratio (*p* < 0.01) decreased. There was no statistical difference in the waist/hip ratio (*p* > 0.05).

At the end of the 12^th^ week, the following parameters were significantly lower in the synbiotic group compared to the placebo group: body weight (*p* < 0.01), body weight Z-score (*p* < 0.05), BMI (*p* < 0.05), BMI Z-score (*p* < 0.01), waist circumference (*p* < 0.05), waist circumference-height ratio (*p* < 0.05). There was no statistical difference between the other anthropometric parameters after 12 weeks.

### Comparison of Laboratory Parameters

At baseline, there was no statistically significant difference between the two groups for glucose and lipid parameters (*p* > 0.05). There was no statistical difference in serum glucose, insulin, HOMA-IR, AST, ALT, total cholesterol, triglyceride, HDL and LDL values in the synbiotic and placebo groups at baseline and after 12 weeks of intervention (*p* > 0.05). Similarly, the percentage of patients with dyslipidemia, mean systolic and diastolic blood pressure values, and the presence of NAFLD were found to be similar between the groups at the beginning of the study and at the end of the 12th week (*p* > 0.05) (Table 2). The percentage of patients with NAFLD were found to be similar between the synbiotic and placebo group at the beginning of the study (14/30, 46.6% vs. 14/31, 45.1%; *p* > 0.05) and also at the end of the 12th week (12/30; 40% vs. 14/31; 45.1%; *p* > 0.05). No adverse events have been reported during the study period related with synbiotic or placebo group.

**Table 2:** Evaluation of biochemistry and lipid parameters of the synbiotic and placebo groups at the beginning of the study and at the end of the 12th week.

**Parameters**	**Synbiotic group** ***n*** **=** **30**		**Placebo group** ***n*** **=** **31**		**p**
	**Baseline**	**12^**th**^ Weeks**	**Baseline**	**12^**th**^ Weeks**	**ns**
Glucose (mg/dl)	83.4 ± 6.12	86.0 ± 6.68	81.6 ± 5.24	83.9 ± 7.48	ns
Insulin	20.2 ± 10.6	17.5 ± 8.12	19.9 ± 11.8	17.1 ± 7.64	ns
HOMA-IR	4.23 ± 2.29	3.69 ± 1.73	4.32 ± 2.93	3.60 ± 1.72	ns
AST (IU/L)	23.6 ± 11.1	21.3 ± 7.0	21.0 ± 5.0	20.0 ± 4.1	ns
ALT (IU/L)	26.6 ± 18.5	26.1 ± 7.7	23.9 ± 13.2	20.5 ± 8.6	ns
Total cholesterol (mg/dl)	161.6 ± 28.9	163.1 ± 30.0	158.1 ± 37.8	157.3 ± 35.4	ns
HDL-C (mg/dl)	43.7 ± 7.1	43.4 ± 6.1	44.5 ± 11.9	44.8 ± 10.4	ns
LDL-C(mg/dl)	108.3 ± 24.7	108.8 ± 28.5	106.8 ± 35.0	101.0 ± 35.4	ns
Triglyceride (mg/dl)	128.1 ± 49.0	121.1 ± 62.0	142.5 ± 91.6	104.7 ± 95.2	ns
Presence of NAFLD (%, *n*)	46.% (14/30)	40% (12/30)	45.1% (14/31)	45.1% (14/31)	ns

*The data all data were expressed as mean ± standart deviation, except the presence of NAFLD has been shown as percentage. There is no statistical significance between baseline and 12^th^ weeks results and also between synbiotic and placebo group. AST, aspartate aminotransferase; ALT, alanine aminotransferase; HDL-C, high-density lipoprotein-cholesterol; LDL-C, low-density lipoprotein-cholesterol; HOMA-IR, homoeostatic model assessment for insulin resistance; NAFLD, non-alcholic fatty liver disease*.

## Discussion

In this study, daily multispecies synbiotic intake, along with diet and exercise, had a more substantial favorable effect on anthropometric parameters (body weight, BMI, waist circumference, and waist/height ratio) in children and adolescents with exogenous obesity than placebo. A significant reduction in (i) body weight of 4%, (ii) BMI of 5.1 percent, (iii) waist circumference of 6%, and (iv) hip circumference of 2.4 percent was achieved after 12 weeks of synbiotic use.

In our clinic between 2011 and 2012, the efficacy of the identical synbiotic mixture was tested in children with exogenous obesity during a one-month intervention (not placebo controlled, single blind research) (13). After therapy, 71.4 percent of the adolescents in the synbiotics group lost weight, and body weight, body mass index, and triceps skinfold thickness values all decreased statistically significantly (13). In this new study, when compared to the trial baseline, the synbiotic group had a significant drop in body weight (all patients), body weight Z-score, BMI, BMI Z-score, triceps skinfold thickness, upper arm circumference, waist and hip circumference, and waist-to-height ratio at the conclusion of the 12^th^ week. The fact that synbiotic therapy was provided for 3 months in the new trial explains the better results when compared to our prior study using the same preparations. When the synbiotic group and the placebo group were compared at the end of the 12^th^ week, it was discovered that the synbiotic group had a greater percent decrease in body weight, percent decrease in BMI value, decrease in BMI Z-score, decrease in waist circumference, and decrease in waist circumference-height ratio. The considerable reduction in waist circumference, which is directly linked to cardiovascular risk, is quite noteworthy (17).

Perna et al. (7) included 20 randomized controlled trials in their study of the effectiveness of probiotics for the management of body weight and anthropometric parameters in adults (*n* = 1,411) with overweight and obesity (7). Despite no substantial reduction in body weight, probiotic administration was found to have a favorable effect on BMI (6). Another comprehensive analysis of the benefits of probiotics and synbiotics on weight loss in overweight and obese people found that 23 of 27 trials showed positive results in terms of weight loss and other anthropometric measurements (6). The administration of biotics was commonly combined with energy restriction and increased physical activity (11 studies), like in our investigation. In these studies that reveal favorable effects of pro- and synbiotics on anthropometric parameters (6, 12). Twenty-four of the 27 investigations were undertaken in adult populations, while three were conducted in children (6). Two lactobacilli (*L. acidophilus and L. rhamnosus*) and two bifidobacteria strains were included in the synbiotic formulation evaluated in our investigation (*B. bifidum and B. longum*). The combination of Lactobacillus and Bifidobacterium had a favorable effect on body weight loss in subjects who also maintained their usual lifestyle (9). There are few studies on the effects of probiotics and synbiotics in the treatment of childhood obesity (8). In a meta-analysis research involving nine randomized trials from Iran, Italy, Turkey, Denmark, Spain, and the United States, Mohammadia et al. evaluated the effects of probiotic and synbiotic use on anthropometric and metabolic markers in overweight and obese children and adolescents (8). They examined data from 410 kids in the study (215 probiotic/synbiotic, 195 controls) (six probiotic and three synbiotic studies). The usage of probiotics for 4–16 weeks had no statistically significant impact on body weight, BMI Z-score, hip circumference, blood sugar, or lipid markers in overweight and obese children, according to this meta-analysis (8). Subgroup analyses, on the other hand, revealed that synbiotics had an influence on the BMI Z-score (8). When compared to a placebo group, children receiving synbiotics had a lower BMI Z-score and higher levels of cytokines and adiponectin, and synbiotic supplementation was expected to have a favorable effect on inflammation (18).

One of the most frequent liver illnesses in children is non-alcoholic fatty liver disease (16). Recent research suggests that the interaction between the liver and the gut, known as the “gut-liver axis,” may play a key role in the phenotypic flip from NAFLD to a more aggressive liver disease such as non-alcoholic steatohepatitis (NASH) and NASH-related fibrosis. Dysbiosis has been linked to the development of NAFLD in children in recent research (19, 20). As a result, numerous writers have proposed modulating gut microbiota with pre-/pro-/synbiotics as a potential treatment for obesity-related NAFLD (21–24). At the start of the study and at the end of the 12th week, the percentage of patients with NAFLD was found to be similar in the synbiotic and placebo groups. We also found no effect of probiotics and synbiotics on glucose and lipid metabolism. This could be linked to the intervention's short duration. Probiotics can affect the lipid profile and insulin sensitivity, two processes that can improve body weight, BMI, waist, and hip circumference (6–9). Probiotics have been proven to lower total cholesterol, triglycerides, and LDL-C levels while increasing HDL-C levels (7, 25).

The gut microbiota may influence whole-body metabolism through influencing energy balance, glucose metabolism, and low-grade inflammation linked to obesity and metabolic diseases. The effect mechanisms of pre/pro/synbiotics on preventing weight gain or loss in obesity have been the subject of numerous hypotheses. Reduction of inflammation, strengthening of the intestinal epithelial barrier, prevention of bacterial translocation, modulation of intestinal enzyme activity, effects on neuroendocrine and immunological functions, inhibition of energy storage and food intake, reduction of dietary cholesterol absorption, prevention of bile acid reabsorption in the small intestines, and reduction of intestinal inflammation are some of the benefits. The association between the microbiome and obesity is complicated, and many factors remain unknown (4, 6). The effects of probiotics and synbiotics on the composition of the gut microbiota are strain-specific.

A limitation of our study is that the outcome is based on the patients' reporting on dietary compliance and exercise recommendations. Despite our patients' great compliance, the majority of those who were enrolled were unable to visit our clinic due to mitigation strategies (stay-at-home orders or hospital reorganization) implemented during the first year of the COVID-19 epidemic. According to International Scientific Association of Probiotics and Prebiotics (ISAPP), studies on a “synergistic synbiotic” that compare the synbiotic to the control can provide supportive evidence but do not constitute a direct evidence that confirms a synergistic effect. Instead, a study including the combination, the substrate alone, the live microorganisms alone and a control should be conducted (8).

## Conclusion

This randomized placebo controlled study found that taking a specific synbiotic for 12 weeks in addition to dietary and physical activity recommendations had a positive effect on anthropometric measurements, resulting in a 4 percent reduction in body weight, 5.1 percent reduction in BMI, 6 percent reduction in waist circumference, and 2.4 percent reduction in hip circumference. Twelveweeks use of synbiotics have some beneficial effects of anthropometric measurements, and these effects might be explained potential effects of synbiotics on microbiota composition. In childhood obesity, the administration of this specific multistrain synbiotics is an effective weight-loss method in addition to diet and exercise.

## Data Availability Statement

The raw data supporting the conclusions of this article will be made available by the authors, without undue reservation.

## Ethics Statement

The studies involving human participants were reviewed and approved by Eskisehir Osmangazi University Faculty of Medicine Local Ethical Committee. Written informed consent to participate in this study was provided by the participants' legal guardian/next of kin.

## Author Contributions

GK, MD, YV, and ED conceptualized and designed the study. GK recruited participants and collected samples. ED interpreted the analyses. All authors contributed to drafting and critical review of the manuscript.

## Funding

This study was financially supported by Eskisehir Osmangazi University Research Grant (201911046). The synbiotic and placebo were supplied by Nobel Ilac, Turkey and Cell Biotech Co., South Korea; both companies had no role in the study design, laboratory analysis and interpretation of the results, preparation, and review of the manuscript.

## Conflict of Interest

YV has participated as a clinical investigator, and/or advisory board member, and/or consultant, and/or speaker for Abbott Nutrition, Ausnutria, Biogaia, By Heart, CHR Hansen, Danone, ELSE Nutrition, Friesland Campina, Nestle Health Science, Nestle Nutrition Institute, Nutricia, Mead Johnson Nutrition, Phathom Pharmaceuticals, Pileje, United Pharmaceuticals Novalac, Yakult, Wyeth. ED has participated as a advisory board member, and/or consultant, and/or speaker for Biocodex, Nutricia, Nestle Health Science. The remaining authors declare that the research was conducted in the absence of any commercial or financial relationships that could be construed as a potential conflict of interest.

## Publisher's Note

All claims expressed in this article are solely those of the authors and do not necessarily represent those of their affiliated organizations, or those of the publisher, the editors and the reviewers. Any product that may be evaluated in this article, or claim that may be made by its manufacturer, is not guaranteed or endorsed by the publisher.
